# 24-Hour Holter Monitoring for Identification of an Ideal Ventricular Rate for a Better Quality of Life in Atrial Fibrillation Patients

**DOI:** 10.7759/cureus.37181

**Published:** 2023-04-05

**Authors:** Zulkefli Sanip, Zurkurnai Yusof, Ng Seng Loong, Nyi Nyi Naing, W. Yus Haniff W. Isa

**Affiliations:** 1 Central Research Laboratory, School of Medical Sciences, Universiti Sains Malaysia, Kubang Kerian, MYS; 2 Department of Internal Medicine, School of Medical Sciences, Universiti Sains Malaysia, Kubang Kerian, MYS; 3 Cardiology Department, Perak Community Specialist Hospital, Ipoh, MYS; 4 Community Medicine Unit, Faculty of Medicine, Universiti Sultan Zainal Abidin, Kuala Terengganu, MYS

**Keywords:** ventricular rate, quality of life, holter, electrocardiography, atrial fibrillation

## Abstract

Introduction

Atrial fibrillation (AF) is the most common persistent cardiac arrhythmia. The impact of AF on quality of life (QoL) is significant, and much has related to the achieved resting ventricular rate (VR). Strategies to control VR can improve QoL in AF patients. However, the ideal VR target remains unclear. Therefore, we aimed to identify the ideal VR target by comparing the QoL of AF patients with different VR cut-off means from the 24-hour Holter (Holter).

Methods

A cross-sectional study was conducted on AF patients in the international normalized ratio (INR) clinic at Hospital Universiti Sains Malaysia. Patients were fixed with a Holter monitor while QoL was measured using the SF-36v2 Health Survey. Patients were repeatedly divided into mean 24-hour Holter VR above and below 60, 70, 80, 90, and 100 beats per minute (bpm). The differences in the total SF-36v2 score and its components were examined.

Results

A total of 140 patients completed the study. There was a significant difference in physical role, vitality, mental health, mental component summary, and total SF-36v2 scores for VR above and below 90 bpm. The total SF-36v2 score difference was also significant in the covariate analysis, while other VR cut-offs (60, 70, 80, and 100 bpm) did not show significant changes in total SF-36v2 scores.

Conclusion

Significant differences were observed in the QoL scores among AF patients, with a cut-off VR of 90 bpm favoring patients with the higher rate. Hence, higher VR is better in terms of QoL among stable AF patients.

## Introduction

Quality of life (QoL) is an important parameter in managing atrial fibrillation (AF) patients, and ample evidence demonstrates that AF is associated with impaired QoL [1]. AF is the most common persistent cardiac arrhythmia. AF symptoms contributed to the higher risk of hospitalization, which led to reduced QoL among AF patients. QoL was also influenced by the severity of the AF symptoms [2]. Over a year, one out of every five older adults with AF experienced a significant decline in QoL [3]. Meanwhile, the prevalence of poor sleep quality increased with the severity of AF symptoms [4]. Thus, it is critical to manage the AF in order to restore high QoL in AF patients.

Controlling the ventricular rate (VR) is imperative to managing AF, thus improving QoL. In the first Rate Control versus Electrical Cardioversion for Persistent Atrial Fibrillation (RACE) study, QoL in uncontrolled AF was significantly reduced [5], while rate or rhythm intervention exhibited an improvement on a health-related QoL survey [1,6-8]. However, the initial step is to determine the best rate to aim for in patients with AF, which is still unclear. So far, estimates have been based on observational studies and experts' consensus [9-11].

The current guidelines recommend a target heart rate of ≤80 beats per minute (bpm) at rest. However, previous evidence recommended that a heart rate control strategy targeting a lenient heart rate (resting heart rate of <110 bpm) is not inferior to a strict heart rate (resting heart rate of <80 bpm) in terms of mortality and hospitalization [12]. Meanwhile, the second RACE study found that in permanent AF patients, the main study endpoints (a composite of clinical events, heart failure classification, and hospitalizations) were not significantly different between the strict and lenient groups [13-15]. In addition, both groups' AF symptoms and QoL were similar [15]. Hence, the recognition of lenient heart rate as an alternative target. Targeting a lenient heart rate was easier to achieve with the same effectiveness as a strict heart rate strategy [12]. 

There is a lack of studies comparing QoL in a population of stable AF patients with multiple ventricular rate targets using the 24-hour Holter monitor (Holter). The usefulness of 24-hour Holter monitoring in detecting arrhythmias among elderly heart failure patients has been reported before [16]. While there is evidence that rate-control strategies improve QoL in AF patients [1,6-8], the best rate to aim for and its relationship to QoL indicators remain unclear. Therefore, in this study, we used multiple cut-off ventricular rates (VR) of 60, 70, 80, 90, and 100 bpm based on a 24-hour mean VR by Holter monitoring. The aim was to identify the best target VR by comparing the QoL scores of AF patients divided by the cut-off rate. This will add more evidence to the ideal resting heart rate, especially among stable AF patients receiving outpatient treatment.

## Materials and methods

This cross-sectional study was conducted on AF patients in the international normalized ratio (INR) clinic at Hospital Universiti Sains Malaysia. The study protocol has been approved by the Research Ethics Committee (Human) of Universiti Sains Malaysia with reference number USMKK/PPP/JEPeM [234.3.(03)] and has been conducted following the principles described in the Helsinki Declaration. All patients have given their informed consent for participation in the study. The inclusion criteria were patients with any documented type of AF, age >18 years, and a resting heart rate of <110 bpm by manual pulse palpation. The exclusion criteria were a history of previous catheter ablation, Wolf-Parkinson-White syndrome, and the use of a pacemaker. Approximately 20 AF patients visit the INR clinic every week. The first three patients who fulfilled the criteria were recruited weekly until the desired sample size was reached. The type, symptoms, European Heart Rhythm Association (EHRA) score of AF, New York Heart Association (NYHA) classification for heart failure, and current medication were all documented.

A study nurse fixed a Holter recorder and administered the SF-36v2 Health Survey. The Mortara System Version H3 24-hour Holter ambulatory monitor (Mortara Instrument Inc., Milwaukee, USA) was used in this study. SF-36v2 was licensed (CT130658/OP011556) to the corresponding author from Quality Metric Inc. USA (2011), and the local language version was used. The SF-36v2 measured the score of the physical and mental components, which consisted of physical functioning (PF), physical role (PR), bodily pain (BP), general health (GH), vitality (VT), social functioning (SF), emotional role (ER), mental health (MH), physical component summary (PCS), and mental component summary (MCS). The score for each component ranged between 0 and 100, while the maximum score for the ten components was 1000. The total SF-36v2 score was calculated by summing up all the components of the questionnaire. The higher score indicated better QoL and vice versa. Using a Holter mean VR of 60, 70, 80, 90, and 100 bpm, the patients were divided into two groups, starting with those above and below 60 bpm, and analyzed. Then they will be divided again into the different Holter VR cut-off means. The process was repeated until the cut-off mean VR of 100 bpm was reached. Following that, the patients did not return unless a new or dangerous arrhythmia developed.

Statistical analysis

The data was analyzed using the IBM® SPSS® Statistics for Windows, Version 26.0 (IBM® Corp., Armonk, USA). Continuous variables were presented as the mean (standard deviation - SD) or median (interquartile range - IQR), while categorical variables were presented as numbers (percentages). The mean difference for continuous variables was analyzed using the independent t-test or Mann-Whitney U test, whereas the Chi-square test (χ^2^) or Fisher's exact test was performed for categorical variables. Analysis of covariance (ANCOVA) with the Bonferroni correction method was executed to compare the total SF-36v2 scores between the groups with an adjustment for potential confounders (age, weight, hypertension, ischemic heart disease, history of stroke, chronic obstructive pulmonary disease/asthma, obstructive sleep apnea, and non-ischemic cardiomyopathy) in the study. Differences exhibiting p-values below 0.05 were considered significant.

## Results

A total of 140 patients completed the Holter recordings. The mean age of the total population was 62.1 (SD=9.37) years, ranging from 26 to 82 years. Most (81.4%) of the patients were aged ≥55 years, with 62 (44.3%) patients being over 65 years old. The youngest patient was a 27-year-old female with mitral stenosis, while the oldest patient was an 82-year-old female with hypertension. The ratio of male to female patients was 1.3:1. The median duration of AF for all patients was 58 months (range of 22-119), and the mean INR level was 2.35 (SD=1.4). In this study, the first detected AF and long-standing persistent AF types were not documented since the study population already had an established timeline from the date of diagnosis. Moreover, none had been subjected to any rhythm conversion attempt after one year. Hence, only permanent, paroxysmal, and persistent AF types were recorded. Most were permanent AF patients, followed by paroxysmal and persistent AF (Table 1).

**Table 1:** The characteristics of study patients according to the cut-off VR of 90 bpm For concurrent medical conditions, one patient may have more than one condition. This is also applied to types of medications prescribed to the patients. Other conditions - two previous pericardial effusions; one obstructive sleep apnea; five asthma/COPD; two non-ischemic cardiomyopathies; two old PTB; one sinus node disease. *Chi-square test. VR - ventricular rate; AF - atrial fibrillation; SD - standard deviation; IQR - interquartile range; CKD - chronic kidney disease; IHD - ischemic heart disease; CRHD - chronic rheumatic valvular heart disease; VSD - ventricular septal defect; ASD - atrial septal defect; COPD - chronic obstructive pulmonary disease; PTB - pulmonary tuberculosis

Patient's clinical characteristics	Total (n=140)	<90 bpm (n=118)	>90 bpm (n=22)	p-value
Age (years), mean (SD)	62.1 (9.37)	62.4 (9.78)	60.2 (6.65)	0.301
Male sex, n (%)	79 (56.4)	65 (55.1)	14 (63.6)	0.458
Duration of AF (month), median (IQR)	58 (22-119)	104 (21-125)	60 (30-90)	0.766
Paroxysmal AF, n (%)	34 (24.3)	34 (28.8)	0 (0.0)	0.004^*^
Persistent AF, n (%)	17 (12.1)	17 (14.4)	0 (0.0)	0.074
Permanent AF, n (%)	89 (63.6)	67 (56.8)	22 (100)	<0.001^*^
Previous heart failure, n (%)	80 (57.1)	69 (58.5)	11 (50.0)	0.461
Previous stroke, n (%)	32 (22.9)	28 (23.7)	4 (18.2)	0.569
Diabetes mellitus, n (%)	37 (26.4)	31 (26.3)	6 (27.3)	0.922
Hypertension, n (%)	112 (80.0)	94 (79.7)	18 (81.8)	>0.995
IHD, n (%)	38 (27.1)	31 (26.3)	7 (31.8)	0.591
Hyperlipidemia, n (%)	94 (67.1)	78 (66.1)	16 (72.7)	0.544
CKD, n (%)	5 (3.6)	5 (4.2)	0 (0.0)	>0.995
CRHD, n (%)	49 (35.0)	43 (36.4)	6 (27.3)	0.408
Thyroid disorders, no. (%)	20 (14.3)	17 (14.4)	3 (13.6)	>0.995
Congenital heart, n (%) (1 VSD; 3 ASD)	4 (2.9)	4 (3.4)	0 (0.0)	>0.995
Other conditions, n (%)	13 (9.3)	10 (8.5)	3 (13.6)	0.430

Most of the patients had EHRA scores of I and II. The primary rate-control agents were beta-blockers and digoxin, whereas amiodarone, diltiazem, and verapamil were used in a small number of patients. Most patients were already on ACEi/ARB and statins (Table 2).

**Table 2:** Clinical data of the study patients, according to the cut-off VR of 90 bpm *Independent t-test. VR - ventricular rate; ACEi - angiotensin-converting-enzyme inhibitor; ARB - angiotensin receptor blockers; EHRA - European Heart Rhythm Association; EF - ejection fraction; NYHA - New York Heart Association; diuretics include frusemide, thiazides and indapamide

Clinical data and list of medications	Total (n=140)	<90 bpm (n=118)	>90 bpm (n=22)	p-value
EHRA-current score, n (%)				
EHRA I	90 (64.3)	74 (62.7)	16 (72.7)	0.368
EHRA II	44 (31.4)	38 (32.2)	6 (27.3)	0.647
EHRA III	6 (4.3)	6 (5.1)	0 (0.0)	0.590
Echocardiography				
EF by Teichholz (%)	62.6 (9.1)	63 (9.1)	59 (8.7)	0.061
Left atrium size (mm)	43.8 (8.3)	43.6 (8.7)	44.4 (6.0)	0.712
NYHA scores, n (%)				
NYHA I	77 (55.0)	66 (55.9)	11 (50.0)	0.608
NYHA II	59 (42.1)	49 (41.5)	10 (45.5)	0.732
NYHA III	4 (2.9)	3 (2.5)	1 (4.5)	0.605
Weight (kg)	67.8 (21.0)	65.8 (20.7)	78.6 (19.8)	0.008^*^
Systolic blood pressure (mmHg)	132 (16)	132 (16)	131 (14)	0.992
Diastolic blood pressure (mmHg)	75 (13)	74 (13)	80 (12)	0.029^*^
Holter data				
Mean VR	76 (13)	72 (9)	98 (7)	<0.001^*^
Minimum VR	46 (9)	45 (8)	53 (9)	<0.001^*^
Maximum VR	136 (30)	130 (28)	167 (20)	<0.001^*^
Percentage of AF	67 (41)	61.9 (43.2)	93.1 (7.0)	0.001^*^
Longest R-R interval (sec)	2.39 (0.69)	2.44 (0.72)	2.12 (0.35)	0.043^*^
Medications, n (%)				
Beta-blockers	62 (44.3)	52 (44.1)	10 (45.5)	0.904
Digoxin	44 (31.4)	37 (31.4)	7 (31.8)	0.966
Amiodarone	4 (2.9)	4 (3.4)	0 (0)	>0.995
Diltiazem/Verapamil	6 (4.3)	6 (5.1)	0 (0)	0.590
ACEi/ARB	84 (60.0)	71 (60.2)	13 (59.1)	0.924
Statin	105 (75.0)	89 (75.4)	16 (72.7)	0.789
Diuretics	62 (44.3)	55 (46.6)	7 (31.8)	0.200

No significant difference was noted between the SF-36v2 components at the cut-off VR of 60 and 70 bpm. VT, MH, and MCS scores were significantly higher at the cut-off VR of above 80 bpm. The MH score was significantly higher at the cut-off VR above 100 bpm (Table 3).

**Table 3:** The difference between SF-36v2 components according to the cut-off VR at below and above 60, 70, 80, and 100 bpm *Independent t-test, p<0.05, below vs. above cut-off VR - ventricular rate, PF - physical functioning; PR - physical role; BP - bodily pain; GH - general health; VT - vitality; SF - social functioning; ER - emotional role; MH - mental health; PCS - physical component summary; MCS - mental component summary

SF-36v2 components	<60 bpm (n=131) vs. >60 bpm (n=9)	p-value	<70 bpm (n=42) vs. >70 bpm (n=98)	p-value	<80 bpm (n=96) vs. >80 bpm (n=44)	p-value	<100 bpm (n=134) vs. >100 bpm (n=6)	p-value
PF	52.8 (28.5) 54.1 (24.1)	0.873	54.2 (23.7) 54.0 (24.6)	0.967	53.0 (23.5) 56.4 (26.0)	0.444	53.5 (24.1) 65.8 (27.8)	0.225
PR	52.8 (24.0) 55.1 (23.3)	0.778	50.6 (21.7) 56.8 (23.8)	0.152	52.5 (23.8) 60.2 (21.4)	0.068	54.6 (23.2) 61.5 (27.2)	0.484
BP	57.2 (20.2) 59.6 (23.3)	0.763	58.1 (23.8) 60.0 (22.8)	0.657	58.9 (23.4) 60.8 (22.5)	0.641	59.1 (23.1) 68.2 (19.7)	0.346
GH	51.0 (20.5) 54.3 (18.2)	0.601	55.0 (19.4) 53.7 (17.9)	0.689	54.4 (18.5) 53.4 (18.0)	0.748	53.6 (18.2) 65.8 (18.6)	0.108
VT	52.1 (16.8) 57.8 (17.9)	0.353	55.8 (17.5) 58.2 (18.1)	0.476	55.1 (18.0) 62.5 (16.7)	0.023*	57.0 (17.9) 67.7 (13.9)	0.151
SF	73.6 (32.7) 71.5 (20.2)	0.769	68.5 (24.3) 73.0 (19.5)	0.247	70.2 (22.2) 74.7 (18.2)	0.238	71.3 (21.2) 79.2 (17.1)	0.370
ER	67.6 (20.6) 64.2 (24.2)	0.687	60.7 (21.9) 66.1 (24.7)	0.227	62.9 (24.2) 67.8 (23.3)	0.266	64.3 (24.2) 68.0 (19.3)	0.709
MH	67.2 (24.9) 72.2 (16.1)	0.392	70.0 (18.8) 72.7 (15.8)	0.392	69.0 (17.3) 78.2 (13.4)	0.002*	71.1 (16.6) 89.2 (10.7)	0.009*
PCS	38.9 (10.6) 39.9 (8.7)	0.731	39.8 (9.1) 39.9 (8.7)	0.940	39.8 (8.5) 40.1 (9.3)	0.842	39.7 (8.7) 43.1 (9.8)	0.360
MCS	47.0 (8.8) 47.7 (8.5)	0.801	46.1 (8.0) 48.4 (8.6)	0.146	46.4 (8.6) 50.5 (7.6)	0.007*	47.4 (8.5) 53.4 (5.5)	0.091
Total score	560.2 (140.9) 576.5 (146.8)	0.747	558.8 (140.8) 582.6 (148.3)	0.379	562.1 (150.4) 604.6 (132.7)	0.110	571.6 (145.6) 661.9 (139.3)	0.139

Meanwhile, PR, VT, MH, and MCS were significantly higher at the cut-off VR of above 90 bpm. Furthermore, the total SF-36v2 score difference was only significant at the cut-off VR of 90 bpm (Table 4).

**Table 4:** Comparison of SF-36v2 components between below 90 bpm and above 90 bpm groups *Independent t-test, p<0.05, below vs above cut-off CI - confidence interval; PF - physical functioning; PR - physical role; BP - bodily pain; GH - general health; VT - vitality; SF - social functioning; ER - emotional role; MH - mental health; PCS - physical component summary; MCS - mental component summary

SF-36v2 components	<90 bpm (n=118)	>90 bpm (n=22)	Mean difference (95% CI)	t-stat (d*f*)	p-value
PF	52.4 (23.4)	62.7 (27.6)	-10.31 (-21.40, 0.73)	-1.846 (138)	0.067
PR	53.2 (22.7)	64.2 (24.6)	-11.03 (-21.61, -0.45)	-2.061 (138)	0.041*
BP	58.3 (23.0)	65.9 (22.4)	-7.64 (-18.17, 2.89)	-1.434 (138)	0.154
GH	53.5 (18.4)	57.1 (17.9)	-3.55 (-11.95, 4.85)	-0.835 (138)	0.405
VT	55.6 (17.5)	67.6 (16.7)	-12.05 (-20.03, -4.07)	-2.987 (138)	0.003*
SF	70.2 (21.5)	79.0 (17.0)	-8.74 (-18.33, 0.84)	-1.804 (138)	0.073
ER	62.9 (23.5)	72.7 (25.4)	-9.80 (-20.72, 1.12)	-1.775 (138)	0.078
MH	69.9 (16.3)	82.3 (15.2)	-12.36 (-19.78, -4.93)	-3.291 (138)	0.001*
PCS	39.5 (8.4)	42.0 (10.3)	-2.54 (-6.55, 1.48)	-1.249 (138)	0.214
MCS	46.8 (8.3)	52.7 (7.7)	-5.95 (-9.72, -2.19)	-3.124 (138)	0.002*
Total score	562.3 (143.7)	646.2 (140.8)	-83.97 (-149.74, -18.20)	-2.524 (138)	0.013*

Further analysis with the ANCOVA main effect model showed the difference in total SF-36v2 scores was still significant at the cut-off VR of 90 bpm (p=0.020). The adjusted means (95% confidence interval) showed that the VR above 90 bpm maintained higher total SF-36v2 scores as opposed to the VR below 90 bpm (Table 5).

**Table 5:** Comparison of total SF-36v2 scores between below 90 bpm and above 90 bpm groups with and without adjustment for potential confounders *Independent t-test applied ^§^Adjusted mean and mean difference (95% confidence interval), Bonferroni adjustment applied **ANCOVA applied (adjusted for age, weight, hypertension, ischemic heart disease, history of stroke, chronic obstructive pulmonary disease/asthma, obstructive sleep apnea and non-ischemic cardiomyopathy) ANCOVA - analysis of covariance

Ventricular Rate Group	Mean total SF-36v2 score	Mean difference (95% CI)	t-stat (d*f*)	p-value
<90 bpm (n=118)	562.3 (143.7)	83.97 (18.20, 149.74)	2.524 (138)	0.013^*^
>90 bpm (n=22)	646.2 (140.8)			
<90 bpm (n=118)	560.3 (534.22, 586.37)^§^	82.05 (13.15, 150.95)^§^	5.559 (120)	0.020^**^
>90 bpm (n=22)	642.3 (580.05, 704.63)^§^			

## Discussion

In the present study, the MH score was higher at the cut-off VR of 80, 90, and 100 bpm. The cut-off VR of 80 and 90 bpm resulted in higher VT and MCS scores. Meanwhile, the cut-off VR of 90 bpm resulted in a higher PR score. Nevertheless, the difference in the total SF-36v2 score can only be seen at the cut-off VR of 90 bpm. A higher total SF-36v2 score, which indicates better QoL, was notified in the above cut-off VR of the 90 bpm group. Interestingly, even after several potential confounding factors were included in the analysis, the significant changes in the total SF-36v2 score remained.

The background comparison between the cut-off VR of below and above 90 bpm showed differences in body weight, diastolic blood pressure, Holter-percentage of AF, and Holter-longest R-R interval. Higher body weight patients tend to have higher VR and likely better QoL scores. Patients with a VR of above 90 bpm had a higher percentage of AF over 24 hours compared to the below 90 bpm group. It concurred with the type of AF for these patients, who were permanent AF, and they were comfortable with the rate above 90 bpm but below 110 bpm. The lower mean percentage of AF in the below 90 bpm group suggested that some of these patients had a variable frequency of AF episodes. This situation was supported by the fact that all paroxysmal and persistent AF patients (51 out of 118) were included in this group. It was unclear whether a certain type of AF (paroxysmal, persistent, or permanent) would benefit from strict or lenient rate control. However, when permanent AF patients (67 out of 118) were added, we did not see a difference in the QoL score.

At above 90 bpm, the PR, VT, MH, and MCS scores were higher than the below 90 bpm group. All 22 patients in this group had the latest EHRA score of I-II. In a previous study, a higher EHRA score was unfavorable to the patients' health-related QoL [17]. Our patients' QoL scores matched the EHRA AF scores, as a low EHRA score reflects better QoL. This finding indicated that patients with a VR of above 90 bpm were only mildly affected by AF symptoms and had better QoL scores.

VR above 90 bpm resulted in significantly better mental component scores, including VT, MH, and MCS, than VR below 90 bpm, while only PR in physical component parameters showed a significant difference. Other VR cut-offs (60, 70, 80, and 100 bpm) did not record any significant difference in the physical components. In comparison to a previous study among male Brazilian AF patients, the 24-hour Holter monitor VR ≤80 bpm group had a better physical component score than the >80 bpm group. The PF and PSC were significantly different between both groups [6]. In the present study, the VR above 80 bpm and 90 bpm had better QoL scores in mental component parameters (VT, MH, and MCS). This may be explained by the fact that the majority of our patients (63.6%) had permanent AF with a median duration of nearly five years and were on long-term clinic follow-up. Conversely, Jaber et al. did not mention the duration or types of AF [6]. Having more paroxysmal and persistent AF patients would explain the findings of better QoL at strict rate control. However, the relationship between AF type and rate control is unclear. The previous study only included male patients [6], whereas ours included both genders. Almost half (43.6%) of our patients were females. Gender factors in AF may influence QoL in general, as female AF patients had lower QoL or impaired daily activities [17,18]. Studies in Europe and Canada found that female AF patients have a lower QoL than male AF patients [19,20]. The reason for the lower QoL in female AF patients is unknown [13], but some factors, such as the presence of depression symptoms, may play a role [20]. Since female AF patients have a lower quality of life, it is possible that a lenient rate control (<110 bpm) rather than a strict rate control (≤80 bpm) would benefit them. However, more research is required to determine the relationship between gender and the optimal rate of control.

Previous evidence showed that older AF patients with lower cognitive function had lower scores on the mini-mental state examination [21]. However, the possibility of a recurrent minor subclinical stroke cannot be excluded. We postulated that higher VR leads to a negative physical impact in the general population of stable AF patients, and lower VR leads to a negative mental impact. Hence, a balance needs to be struck between higher and lower VR.

This study found the cut-off rate of above 90 bpm in stable AF patients exhibited better QoL, especially in the mental health component. The upper limit is unknown, but our most reliable data is <110 bpm from the second RACE trial. In addition, Jaber et al. [6] reported that a group of patients with a maximal heart rate of ≤110 bpm on a six-minute walk test had a higher score in PF, PCS, GH, VT, MH, and MCS of the SF-36 questionnaire as compared to the >110 bpm group. Hence, the target of <110 bpm should be accepted as the maximum unit. Targeting a strict rate control of <80 bpm for AF patients in the general population was not an easy task. The current study showed 68.6% (96 out of 140 patients) were controlled at below 80 bpm. Meanwhile, in the second RACE trial, 67.3% of AF patients in the clinic achieved the target VR of <80 bpm when electrocardiogram and the 24-hour Holter were used [14]. In our opinion, the proportion of AF patients achieving the target rate should be higher than that. Therefore, a larger number of AF patients will benefit if the target is less stringent, such as from 90 to 110 bpm (lenient rate control). In the long run, the current approach would result in a lower drug burden and cost savings.

Limitation

This study had several limitations. Firstly, using generic QoL tools like the SF-36v2 to quantify the impact of AF on patients' daily lives may not be the best method. A more AF-specific tool, such as the Atrial Fibrillation Effect on QualiTy of Life (AFEQT), could provide more accurate information [22]. Besides that, since SF-36v2 was obtained by self-reporting, the possibility of inaccurate information being given cannot be excluded. However, the gold standard for QoL measurement in AF is still debatable [23,24]. Secondly, this study's distribution of AF types differed from other studies [25,26]. In those studies, the AF types were almost evenly distributed, with permanent and paroxysmal AF accounting for about 30% of the study population and persistent AF accounting for about 20%. The difference was likely because the cross-section of patients in the INR clinic consisted mainly of long-standing stable AF patients. Hence, any suggestions generated by this paper might only be suitable for permanent AF patients. Additionally, only patients with good left ventricle ejection fraction were examined in this study. In patients with a history of heart failure or lower ejection fraction, the AF ventricular rate should be strictly controlled. Moreover, patients were recruited, and Holter recorders were fixed during the clinic's operating hours. For the patients, this could result in white-coat tachycardia. However, as the study used the 24-hour mean heart rate, we anticipate minimal impact from stress tachycardia. Finally, the study only looked at the target VR at rest, not during exertion.

The study showed that in a population of stable AF patients, a VR of less than 80 bpm had lower QoL scores, while patients with higher rates had higher QoL scores. In terms of QoL, VR at or above 90 bpm was the best VR target. This study suggests that it is safe to keep VR above 80 bpm and up to 110 bpm (lenient heart rate) in selected patients.

## Conclusions

In conclusion, better QoL scores were observed in higher mean VR from the 24-hour Holter recording. The cut-off VR above 90 bpm demonstrated a significant difference in QoL, specifically the mental health, vitality, mental components summary, and total Sf-36v2 scores. The findings suggest using a lenient heart rate target (<110 bpm) in managing stable AF patients. Further prospective trials in AF with a different target or a lenient heart rate would be beneficial to confirm this finding.
